# Thermal Aspects in Edge Trimming of Bio-Filled GFRP: Influence of Fiber Orientation and Silica Sand Filler in Heat Generation

**DOI:** 10.3390/ma15144792

**Published:** 2022-07-08

**Authors:** Makram Elfarhani, Fethi Guesmi, Ali Mkaddem, Sami Ghazali, Saeed Rubaiee, Abdessalem Jarraya

**Affiliations:** 1 LA2MP, National School of Engineers of Sfax, University of Sfax, P.O. Box 1173, Sfax 3038, Tunisia; fethiguesmi2017@gmail.com (F.G.); ajarraya@uj.edu.sa (A.J.); 2 Department of Mechanical and Materials Engineering, FOE, University of Jeddah, P.O. Box 80327, Jeddah 21589, Saudi Arabia; amkaddem@uj.edu.sa (A.M.); sghazali@uj.edu.sa (S.G.); 3 Industrial Department, FOE, University of Jeddah, P.O. Box 80327, Jeddah 21589, Saudi Arabia; salrubaiee@uj.edu.sa

**Keywords:** glass fiber, epoxy, silica sand, temperature, edge-trimming, thermographs, heat partition, SEM

## Abstract

The present work aims to determine the influence of Glass Fiber-Reinforced Polymer (GFRP) laminating configuration in heat generation during the dry edge trimming process. Temperature measurement experiments were conducted on pure epoxy matrix, 15% unidirectional glass fiber reinforced epoxy, and 28% silica sand-filled GFRP specimens through eight type-K thermocouples evenly distributed along the trim plans and connected to a data acquisition system. Infrared thermographic measurements were also conducted to investigate the tool temperature evolution while processing. It was found that perpendicular fiber edge milling induces a sharp increase with peak temperature measurements reaching 119 °C, while machining parallel to fiber leads to a maximum temperature history of 41 °C, which is very close to that obtained from the pure epoxy test. It was also found that the addition of silica sand grains in the GFRP matrix reduces both tool and specimen temperature magnitudes up to 67% for 90° plies and 14% for 0° plies compared to silica sand-free composite initial values. The heat partition was calculated from the measured (electric) and estimated energies for the tool, the workpiece, and chips, respectively. It appears from predictions that the addition of silica sand grains increases the heat conductivity of the GFRP materials (with rates of 20% for 0° fiber orientation and 10% for 90° fiber direction), while it reduces that conducted to the milling tool. Scanning Electron Microscopy (SEM) inspections helped detect the dominating machining defects relative to each GFRP configuration and explained the heat generation and dissipation effects in light of peak temperature measurements.

## 1. Introduction

Fiber reinforced polymer (FRP) composite materials are extensively used in many industrial applications, offering attractive properties in terms of low weight, good thermal and chemical resistance, high specific strength, stiffness, and ease of manufacturing, to name a few [1]. These unique advantages have fostered the use of this type of material as the main competitor of metallic materials. In modern cars, for instance, many parts are achieved using only the FRP composites [2,3]. Although the produced surfaces are nearly net-shaped, edge milling is required in the assembling process [4,5]. However, when compared to conventional metals, FRP composites are more troublesome in machining because they are anisotropic and heterogeneous, and especially because of the abrasive and hard-to-cut nature of their fiber reinforcements [6]. Indeed, these materials are manufactured by a combination of mainly two synergistic elements—namely, reinforcement and matrix, with the aim of superposing their superior mechanical properties and addressing the weaknesses of either [7,8]. Therefore, the selected types of matrix and reinforcement materials define the obtained composite material characteristics. In the literature on fibrous composites, the fiber-reinforced thermoset polymer laminates are commonly classified in two types of composite materials: CFRP (Carbon Fiber Reinforced Polymer) and GFRP (Glass Fiber Reinforced Polymer). Historically, glass fibers were the first reinforcements to be industrially developed, and they attract manufacturers for their low cost, good tensile strength, and high deformability [9,10]. Compared to glass fibers, carbon fibers are lighter and stiffer, with higher mechanical and chemical resistances but likewise with a higher cost [11]. Hence, for many GFRP manufacturers, the major benefits of choosing glass fibers for reinforcement of thermosetting resins is their optimum performance-to-cost ratio [12].

Many researchers have discussed the challenges encountered in machining composite materials by addressing four topics: (1) optimization of the cutting parameters [13,14,15,16,17,18], (2) machined surface quality [18,19,20,21], (3) cutting tool life [22,23,24,25], (4) heat partition, and (5) thermal effects [25,26,27]. Indeed, each of these topics treats a side of the possible damage occurring in composite material work-pieces as well as cutting tools during machining. In fact, all of these topics primarily aim to thoroughly understand the correlated phenomena that occur during machining of FRP composite laminates to prevent processing defects, including thermal and mechanical damage to the machined surface and tools used as well [28]. Concerning the first topic, understanding machining parameters has witnessed considerable advances due to the diversity of experiments that were carried out for decades to find the optimum cutting conditions. Many researchers focused on revealing the effects of the spindle speed, the feed rate, and the depth of cut on the other investigation themes. Based on FRP processing tests, researchers argued that the machining force is mainly influenced by the feed rate [13,14] and that it increases when the depth of cut is increased [15]. The vibration analysis technique proposed by Chibane et al. [16] shows that measured vibrations are notably influenced by the interaction of both depth of cut and feed rate. It was also found, through GFRP end milling experiments, that the increase of the depth of cut does not induce any considerable effect on the machined surface roughness [17], while a high spindle speed and low feed rate improve the surface quality [18]. Karpat et al. [19] studied the relationship between the milled CFRP surface and the induced machining force. It was found that keeping a 45° fiber-cutting angle improves the milled surface quality. This result has already been indicated by Hocheng et al., [20] who pointed out that contrary to perpendicular orientation, the best surface qualities are produced when reinforcement fibers are parallel to the feed tool direction. In the same context, Pecat et al. [21] evaluated the quality of milled surfaces for four different fiber orientations through micrographic analyses. It was observed that milling in 0° and +45° leads to smoother surfaces, while serious damage (cracks and segmentations) is incurred when milling in −45° and 90° fiber-cutting angles. On the other hand, several authors give more attention to tool performance and insist that the acceleration of the cutting tool wear is a decisive indication in machining condition adjustments. Faria et al. [22] evaluated the wear resistance of two different tool materials. The authors pointed out that a cemented carbide drill displays lower wear land compared to the high-speed steel one. Kavad et al. [23] showed that machining of GFRP using harder tool materials reduces even the delamination effects in processed parts. Apart from that, the vibration analysis of Chibane et al. [16] showed that tool wear appears at a certain vibration threshold, which is influenced by the interaction of feed rate and depth of cut. Nearly the same results were reached by Lokesh et al. [24]. They deduced, through the analysis of variance of previously conducted polymer composite machining tests, that the use of low feed rate with moderate cutting speed allows for the slowing down of tool wear accelreation. According to Sheikh-Ahmad et al. [25], the hardness of the cutting tool drops when its temperature rises during processing, which causes the acceleration of its wear. Actually, dry machining of FRP materials is more preferred than wet processing conditions [28]. As a result, the increase in cutting temperature is axiomatic and it presents another challenge for researchers and industrialists, since it causes aggressive tool wear and evenly matrix thermal damage. Recent studies [25,26,27] were motivated to introduce the heat partition into the work piece by using different techniques and methods to measure cutting temperature and to estimate their influences on the quality of the machined parts. Liu et al. [27] used the Conjugate Gradient Method, and they pointed out that 21% of the generated heat is evacuated through the composite specimens during milling. Using the inverse heat conduction technique, Sheikh-Ahmad et al. [25] found that the heat partition ratios in the composite sample, chips, and tool were 0.07, 0.37, and 0.56, respectively. In another work [29], the authors deduced that the heat flux conducted through a GFRP edge trimmed surface was more influenced by the feed rate rather than the spindle speed. In summary, the cited studies provided rich qualitative and quantitative correlation analyses between the different FRP machining research topics. Nevertheless, the majority of the available publications focused particularly on the effects of cutting parameters and neglected the influence of composite laminating configuration on thermal aspects during processing. Meanwhile, the thermo-mechanical behavior may affect the quality of the machined surface and the wear condition of the tool as well. As regards the manufacturing configuration, it is widely known that FRP composite materials are manufactured by a combination of reinforcement and matrix in order to superpose their efficient mechanical properties and avoid the weaknesses of either [30,31]. In particular, some industrial experts urge, for environmental, economic, and technical reasons, the addition of silica sand as a filler in the mixture of resin and reinforcement of fibers while manufacturing FRP components [32,33,34]. In fact, this bio filler can ameliorate the mechanical rigidity of the parts and their chemical resistance; it can also reduce the proportion of the thermoset matrix, which is the non-recyclable element in the product. The particularity of this filler lies in its chemical origin, which presents the main component of GFRP reinforcements as most glass fibers are silica based (~50–60% SiO_2_) [35]. This chemical homogeneity between the reinforcements and the fillers can have a great influence on the thermal aspect of GFRP composites during dry machining. Therefore, it is necessary to fully understand the heat generation phenomenon while processing silica sand-filled GFRP composites through specific experiments.

In this work, we propose to conduct a rigorous experimental procedure allowing the evaluation of the influence of each constituent of the composite material on its overall thermal behavior. Especially, the silica sand-filling and the fiber orientation effects were investigated in the light of SEM micrograph analysis of the fresh surface. Another objective was to assess heat transfer between tool, separated chips, and GFRP composites through calculations and measurements of the dissipated heat power of both tool and composite parts.

## 2. Materials and Methods

### 2.1. Specimens Configurations

In this experimental study, we consider five types of composite workpieces: A, B, C, D, and E. Specimen A is a pure epoxy matrix without fiber reinforcement, while specimens B and C were reinforced with glass fibers oriented parallel and perpendicular, respectively, to the assumed tool feed direction. Samples D and E were identical to specimens B and C, respectively, with the addition of silica sand as a filler in the epoxy matrix admixture. Regarding samples D and E, they were identical to specimens B and C, respectively, with the addition of silica sand as a filler in the epoxy matrix admixture. The objective here is to determine the effects of three manufacturing criteria: (i) the presence of fiber, (ii) the orientation of fiber, and (iii) the presence of silicate sand filler and its interaction with the reinforcement fiber orientation. Table 1 shows the volume fraction of each ingredient of the five considered samples. The laminating configurations contained in this table are deduced after several molding trials, and it was found that when the volume fraction of silica sand (with a mean granulometry of 0.5 mm) exceeded 30%, the matrix admixture was very viscous and did not result in good moldings. In addition, below a resin volume fraction of 50%, the laminate strength decreased due to the lack of sufficient epoxy resin to hold the filler grains and the reinforcement fibers together properly [36]. The volume fraction of glass fiber was kept fixed in all stratified samples to eliminate the influence of the reinforcement volume ratio in the experimental results.

#### 2.1.1. Raw Materials

The two epoxy reagents (resin and hardener) belong to RESOLTECH Co., Rousset, France, under the references: 1050 for the resin and 1050 s for the hardener. In Table 2, we summarize the principal characteristics of epoxy resin used for preparing the samples. The reinforcement material was the E-glass unidirectional cloth with a 308 g/m^2^ surface mass provided by Sicomin Co., Châteauneuf-les-Martigues, France. The silicate sand filler was provided by Tecnopol Co., Barcelona, Spain. Table 3 shows the typical characteristics of the filler grains used for preparing samples D and E.

#### 2.1.2. Work-Pieces Preparations

The workpieces were of a rectangular parallelepiped form (80 mm × 40 mm × 8 mm) and made by the compression molding method. The two chemical ingredients that reacted to give the epoxy matrix were mixed manually before molding. The first step of manufacturing consisted of placing ten reinforcement layers impregnated with the matrix mixture in the bottom part of the mold. Then, the mold was closed and subjected to a specific curing cycle recommended by the epoxy manufacturer. This curing cycle involved of leaving the mold at room temperature for 24 h with a uniform pressure of 3 kPa and a maximum humidity level of 70%. Later, the composite laminates within the mold were post-cured for 15 h in autoclave at the same pressure level and a temperature of 60 °C. Finally, the resulting 8 mm-thickness composite plates were laser cut, thus giving multiple samples of every considered type.

### 2.2. Temperature Acquisition System

**Figure 1:**
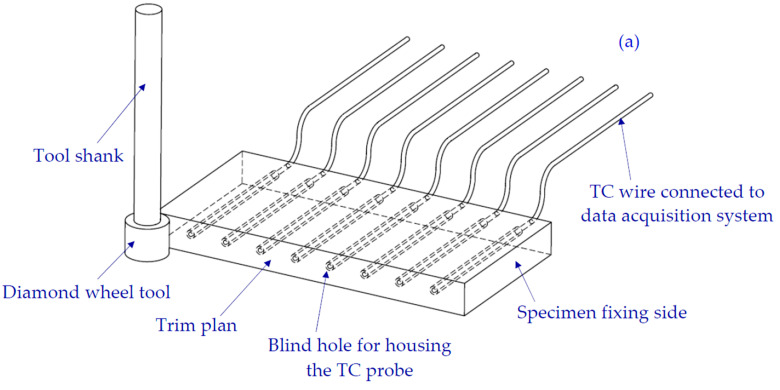

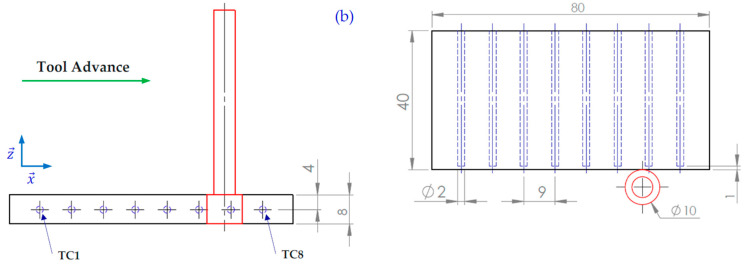
(**a**) Cutting configuration and temperature measurements technic, (**b**) Tool, Sample, and TCs probes holes geometries.

**Figure 2:**
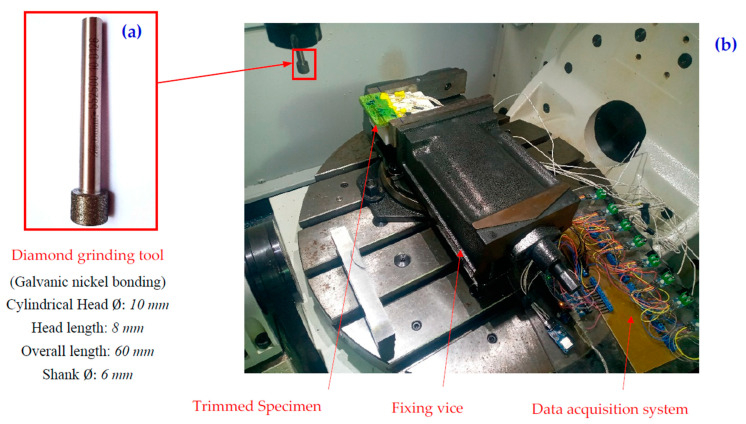
(**a**) Cutting tool configuration, (**b**) Edge milling test set-up.

A temperature acquisition system was specially designed to measure the in-process temperature along the machined surface. A set of eight thermocouples were incorporated into eight pre-drilled blind holes, whose diameter (2 mm) was very slightly larger than that of sensor probes, and evenly distributed along the machining path as illustrated in Figure 1a and Figure 2b. With the intention of minimizing the heat dissipation effect, the sensors were introduced inside the specimen until there was a close distance of 1 mm between the probe tips and the machine surface. The thermocouples (TCs) were of type K (provided by ELECTRONIC SHOP Co., Sfax, Tunisia) and related to an Arduino Mega board, which was connected to the computer via a USB link. The data acquisition process was performed via the PLX-DAQ software interfaced with an Arduino application for processing the signal acquisition and the data transfer. The temperature-sampling rate was set at 4 measures per second. In Table 4, we summarize the technical specifications of each component constituting the temperature acquisition system. Edge milling temperatures were also controlled with an EASIR TM-4 model infrared camera placed at a distance of 70 cm in front of the machined surface. This technique made it possible to control both the increase in the temperature of the tool over the advancement and the decrease in the temperatures of the recently machined parts of the considered surface.

### 2.3. Machining Details

Edge trimming experiments were executed on a Spinner U-620 5-axes High Speed CNC Machine giving a maximum spindle speed of 12,000 rpm and a maximum power of 19 KW. The experimental set-up, including the mounting of the tool, the test piece, and the temperature acquisition device, are shown in Figure 2. The edge trimming operations were performed using a unique diamond grinding-metal bonding tool of 10 mm in diameter, 50 mm in length, and 10 mm of effective cutting length. During processing, the depth of cut and the cutting speed were kept constant at 0.2 mm and 283 m∙mn^−1^, respectively. In these experiments, dry machining was employed since the cutting fluid is not allowed in some industrial fields [38] and is considered disadvantageous to maintaining the chemico-physical characteristics of the epoxy matrix [26].

## 3. Results and Discussion

### 3.1. Temperature Measurement Curves

**Figure 3:**
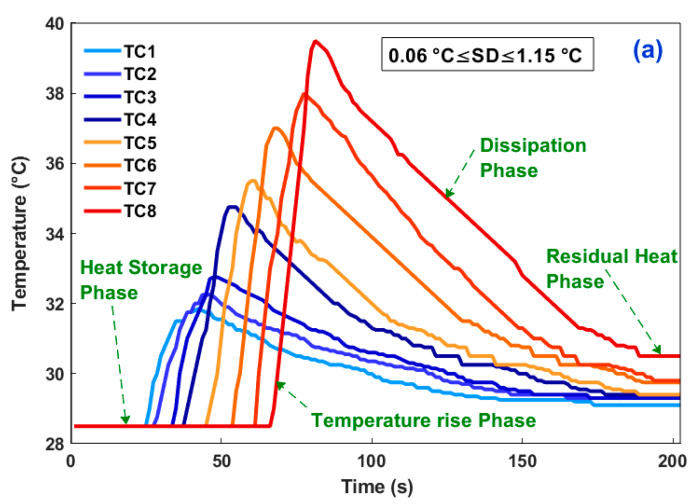

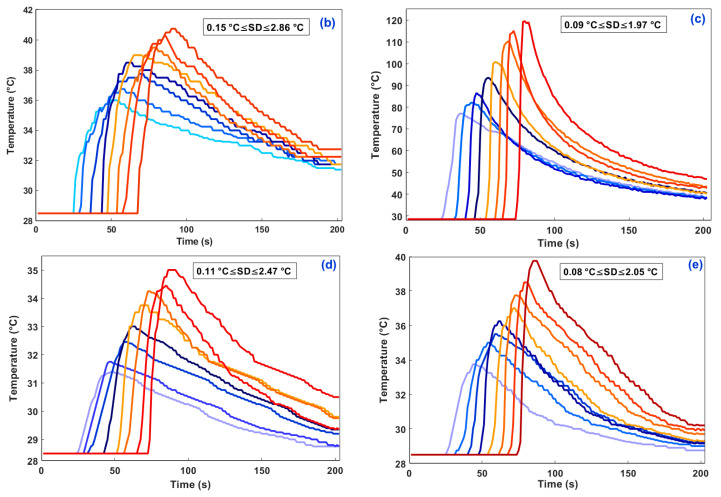
Temperature measurements during machining of the GFRP specimens at a feed rate of 50 mm/min: (**a**) Pure Epoxy matrix, (**b**) 0° GFRP, (**c**) 90° GFRP, (**d**) 0°/silica sand filled GFRP, (**e**) 90°/silica sand filled GFRP.

Signals acquired by the TCs allow the plotting of the temperature history along the trimmed surface. Figure 3 illustrates the obtained curves during machining of the considered work specimens. As indicated in Table 4, the thermocouples provide an accuracy of ±1.5 °C with a 0.25 °C resolution, and from statistical analysis, the standard deviations (SD) were found to be too small and were inserted on Figure 3 as indications [37].

Primarily, this representation of the acquired data shows that the fundamental phenomenon of heat generation, demanganization, and transfer occurred across the milled surfaces. Phenomenologically, three regions can be identified in each plotted measurement (Figure 3a): (1) heat storage region, (2) temperature rise region, and (3) heat dissipation region. Each zone manifests a part of the thermo-mechanical behavior of the composite workpieces facing the cutting conditions. The second zone is particularly troubling in machining since it can spoil the matrix resin and generate internal stresses that can cause it to burst. In this region, the thermocouple measurements exhibited quasi-linear allures over time. Quantifying the heating and cooling rates when the tool passed through the sampling points is necessary to assess the severity of thermal shock. The measurement of the Thermal Diffusion Distance (TDD) likewise appears important. This distance reflects the length of the zone affected by the machining heat just before the tool passes. In Table 5, we summarize the heating rate, the cooling rate, and the TDD values.

Table 5 shows large increases in heating rate values over the course of the process. It is obvious that this is due to the continuous rise of the tool temperature, which presents a storage unit of cumulating heat. Compared to pure epoxy configuration, it is remarkable that the parallel orientation of fibers decreases the heating rate values with a mean ratio of 0.96. Furthermore, the presence of silica sand as a filler favors this decrease with a mean ratio of 0.65. Contrariwise, perpendicular fiber configuration leads to a heavy increase in the heating rate with a mean ratio of 18.86 by reference to that of the pure matrix sample. Similarly, silica sand grains allow the considerable reduction of this heating rate until reaching a ratio of 1.32. Regarding the thermal diffusion distance (*TDD*), it can be noticed that specimen A kept a relatively constant range of this parameter with an average value of 6.12 mm, while sample B yielded the highest mean value (8.94 mm), and specimen C recorded the lowest minimum mean value (3.22 mm). In fact, this parameter gives important indications about the thermal conductivity level along the machined surface. Considering this criterion, it can be seen that heat is conducted more rapidly through 0° fiber orientation specimens, and hardly at all through 90° orientation samples [39]. Otherwise, the influence of the dissipation phase should not be neglected in the dry condition since it causes a thermal shock. For a rapid cooling as an example, the core of the composite material is still hot while its outer part is cold and therefore shrinks. The generated tensile stresses can thus cause cracks or the bursting of the material. Hence, considering the cooling rate as a monitoring parameter can allow the approximate evaluation of the severity of the thermal shock. Graphically, two types of energy dissipation mechanism can be noticed: the first takes a quasi-linear shape (case of samples B, D, and E), while the second takes an exponential form (case of sample C). In any case, having a relatively high thermal dissipation rate indicates that the heat transfer mechanism is harder, which is clear in specimen C and even more so in specimen E.

### 3.2. Peak Temperature vs. Composite Laminating Configuration (Dependencies)

**Figure 4:**
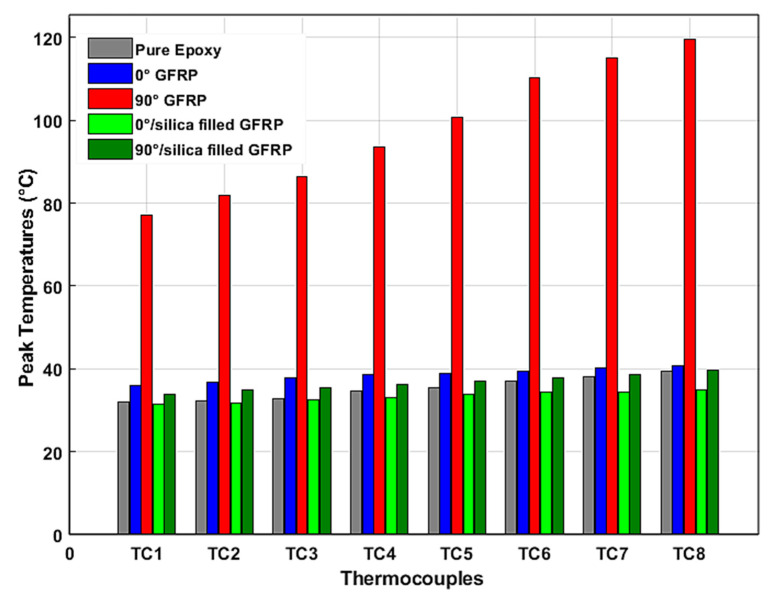
Peak temperatures measurements of each thermocouple (0.08 °C ≤ SD ≤ 2.86 °C).

Among the measurement parameters cited above, the maximum thermocouple output values (peaks) are certainly a good comparison criterion of the specimens’ thermomechanical behaviors. To better perceive and compare the effects of each monitored manufacturing layout in temperature value magnitude, we illustrate in Figure 4 the peak temperature measured by the eight thermocouples (TC1 to TC8) for each planned test in histogram form.

According to the temperature measurement evolution during processing illustrated in Figure 3, and maximum temperature measurements of each specimen shown in Figure 4, we can infer the pure influences of fiber orientation, on the one hand, and the presence of silica sand as filler, on the other hand, on the thermal effects during the edge trimming process.

The comparison of the peak temperature values reached in the considered sampling points reveals that the fiber orientation is very influential in the thermic state of the composite workpiece during edge trimming. Graphically, it can be seen that the perpendicular orientation of reinforcement glass fiber (configuration of specimen C) strongly amplified the peak temperature values (over than 200%) compared to pure epoxy configuration (specimen A). This remarkable temperature magnitude seems to result from the increase of friction forces between the grinding tool and the 90° oriented fibers since many studies, including Henerichs et al. [40], have pointed out that among the different arrangements of reinforcement filaments, fiber orientation of 90° is the most critical orientation as it produces the highest machining forces. However, specimen B (with parallel orientation of fibers) gives at first a slightly higher temperature measurement compared with specimen A. This measurement begins to decrease below that of the pure matrix workpiece starting from the fourth sampling point (TC4). This first observation can be explained by the fact that a parallel orientation of glass fiber does not induce sufficient friction force, which causes an increase of tribology effects and then temperature magnitude [41]. Furthermore, the maintained contact between the tool active area (which is considered here as a moving heat source) and almost the same paralleled filaments of glass fibers allows the relative increase of heat conduction towards the edges of the part. The *TDD* values of Table 5 confirm this hypothesis. In addition, the large contact area between the fibers directly exposed to machining and the following deeper fibers favor a high heat energy absorption. Contrariwise, the difference between peak temperatures while the processing of work pieces C and A continues to increase along the considered machined surface. Here, the heat generation effects caused by the friction between the diamond tool and the perpendicular glass fiber exceeds the absorption phenomenon of reinforcement elements. In fact, we can confirm the phenomenon of heat absorption when comparing the maximum measured temperatures of samples C and E. It is clear that the presence of silica sand as filler in the composite laminate allows the reduction of the temperature magnitude [42]. The same result can be observed when comparing the temperatures of specimens D and B. Comparing the specimens’ peak temperatures with the glass transition temperature (*Tg*) of epoxy matrix can help in valuing the machining thermal severity. In fact, when exceeding the *Tg*, the matrix stiffness dropped, and the rubbery state of the polymer matrix could no longer function effectively to keep the overall composite part strength and to transfer cutting forces to the fibers [43]. Based on the manufacturer’s catalogue, the *Tg* of the used epoxy matrix was 77 °C. From Figure 4, it appears that the edge trimming process of specimen C led to the exceeding of the epoxy matrix *Tg*, while the other samples were below the glass transition limit. The minimum gaps to reach this limit recorded for samples A, B, D, and E were 48.7%, 47%, 54%, and 48.37%, respectively. Hence, it can be deduced that, except for workpiece C, all the laminate configurations are appropriate as their edge trimming process is conducible to an admissible level of temperature rise.

**Figure 5:**
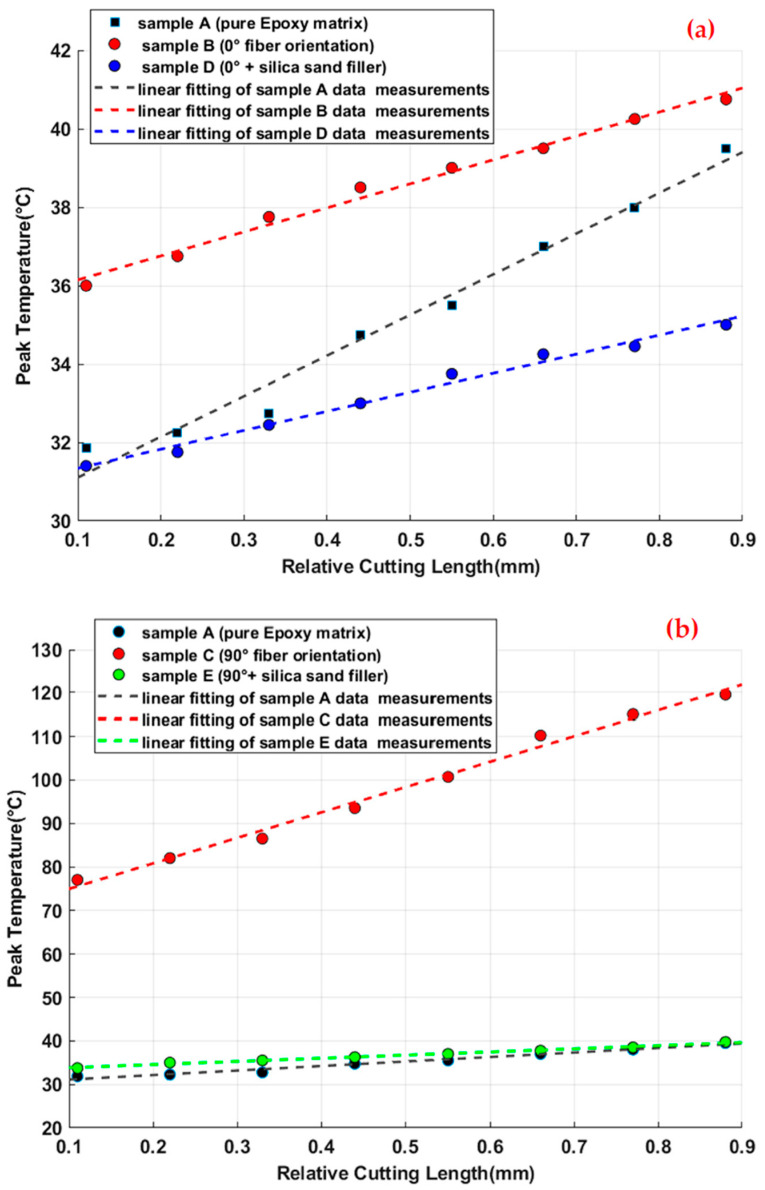
Peak Temperature over relative cutting length: (**a**) 0°fiber orientation samples vs. pure epoxy sample (**b**) 90° fiber orientation specimens vs. pure epoxy specimen.

With the intention of further investigating the heat distribution law throughout the tested work parts, the peak temperature was plotted over relative cutting length (x*_R_*_𝐶_= x_𝑇𝐶_/𝐿_𝐶_) where X_𝑇𝐶_ is the distance at which the *TC* is positioned by reference to the starting point of cutting, and 𝐿_𝐶_ is the total cutting length. Figure 5a,b manifests the plotted data for both fiber orientations by reference to the pure matrix specimens. There is a clear and distinct feature in the plotted peak temperature allures. Primarily, it takes an almost linear shape over time and eventually over the cutting path. Assuming that linear tendencies were reliable enough to reflect the experimental data variation, the law of peak temperature evolution can be represented over time and over cutting length, respectively, as:

(1) $$T_{peak}\left(t\right)=T_{i}+V_{H}\times t$$

(2) $$T_{peak\;}\left(x\right)=T_{i}+\frac{V_{H}}{V_{f}}x$$

(3) $$T_{peak}\left(x_{Rc}\right)=T_{i}+t_{c}\cdot V_{H}\cdot x_{Rc}$$

where *T_i_* is the tool initial temperature, *t_c_* is the total cutting time, *V_f_* is the feed rate, and *V_H_* is the graphical slope of the linear peak temperature evolution reflecting the heating rate.

In Table 6, we give the R-square fitting value and the heating rate (*V_H_*) measurements deduced from the graphical slopes of the peak temperature allure linear fitting as indicated in Equations (2) and (3). According to the provided fit statistics, the R-square values of all fittings are very close to 1. This presents a strong indication that the linear representation considers the major proportions (at least 96%) of peak temperature variance. In fact, these linear allures of peak temperature evolution indicate that temperature increases proportionally with the tool advancement due to the superposition of the generated heat flux. Having a constant heating rate refers principally to the imposed constant feed rate *Vf* (50 mm∙mn^−1^) as it is about a heat transfer at interfaces in the presence of a generated flow and a moving contact. For each test, the diamond tool performs 14,420 revolutions with a feed-per-revolution of 0.0055 mm/rev. Knowing that the contact width is about 1.41 mm, the grinding tool revolutes 256 times in each same contact zone with the machined part. This unicity of the testing condition indicates, as expected, that the difference between the heating rates of the tested parts returns to their laminating configurations since they exhibit different heat dissipation responses.

When comparing the heating rate values of Table 6, it can be clearly deduced that the 0° fiber orientation composite piece manifests heat flux 41% slower than the pure epoxy matrix sample. Contrariwise, the temperature of the 90° fiber orientation part increases over 500% faster than that of reference specimen A. Regarding the influence of silica sand on the heating rate, it allows an 87.7% reduction of the perpendicular fiber orientation composite sample and 19% of the paralleled fiber work part. At this stage, it appears essential to investigate the possibility of approximately anticipating the tool temperature variation history from the specimen’s peak temperature evolution. Experimentally, the plotting of the tool temperature evolution can be verified through infrared thermometric measurements using an infrared camera as detailed in the next paragraph.

### 3.3. Infrared Thermometric Tool Temperature Measurements and Heat Partition Evaluation

**Figure 6:**
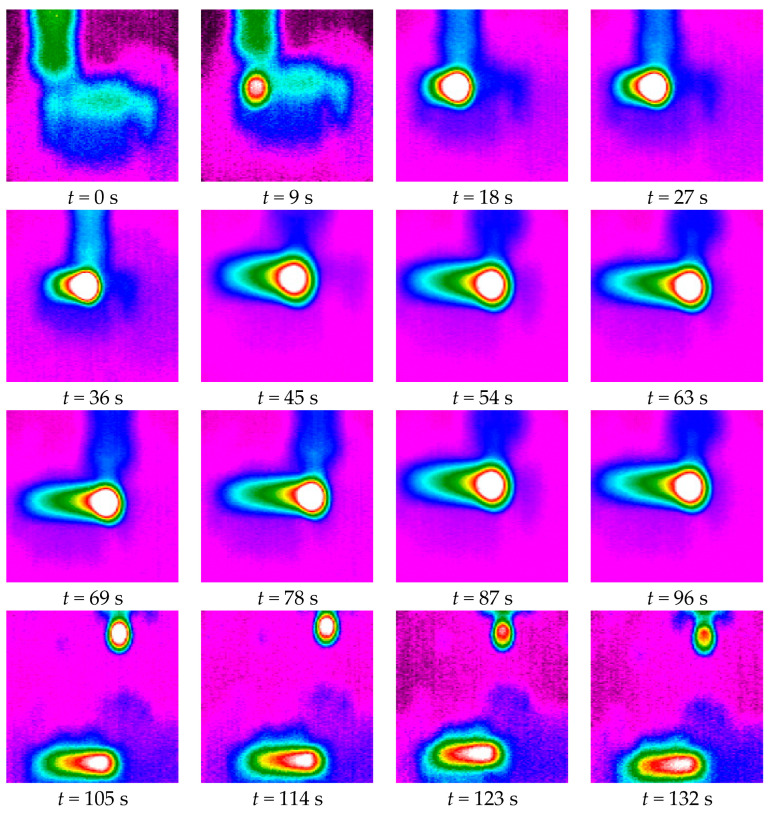
Thermographic shots of the edge trimming process with sampling period of 9 s.

The shots with a thermal camera (EASIR TM-4 model) shown in Figure 6 reveal the heating of the tool-part assembly during and after machining for a sampling period of 9 s. We aim to deduce the evolution law of the tool temperature and to find its correlation with the sample’s laminating configurations.

**Figure 7:**
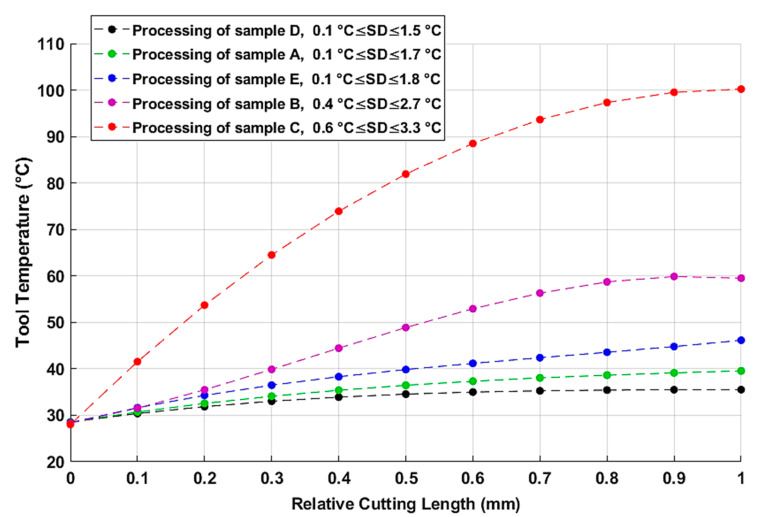
Tool temperature measurements over relative cutting length.

The infrared thermometric measurements allow the plotting of the tool temperature evolution over relative cutting length (with an accuracy range of ±2 °C) as illustrated in Figure 7. Statistical analysis of repetitive measurements gave small error bars which can-not be graphically read; hence, the error ranges were reported on the caption of the plotting. As shown in Figure 7, the tool temperature magnitude is consistent with that of the correspondent processed sample. This proves again that the stratification configuration of the composite material is a primary factor that determines the amount of thermal energy released during the machining process [44]. Indeed, all the measurement plotting presented above shows that silica sand grains play a remarkable role as a filler in reducing the temperature magnitude of both workpiece and tool. In particular, the processing of 0° fiber-oriented GFRP material, filled with silica sand, manifested the lowest heat diffusion level. This is likely attributable to the decrease of the friction effects with the presence of silica sand dust as a lubricator surrounding the active part of the tool and minimizing the effects of dry friction along the cut area.

Based on the temperature evolution measurements of both the grinding tool and machined parts, we can approximate the partition of the generated heat during the edge trimming process. This approach helps us to better understand the thermal aspect of each GFRP laminating configuration. In fact, the heat partition in contact regions can be determined by reference to the following energy balance equation [25]:

(4) $$Q_{Total}=Q_{t}+Q_{w}+Q_{c}=\eta_{e-m}\cdot \eta_{m-h}\cdot P_{e}\cdot ∆t$$

Here, we assume that the net electric power is transformed during the processing time Δ*t*, to a useful mechanic power with a spindle efficiency rate $(\eta_{e-m}=80\%)\;$ and then to heat power with a heat conversion rate $\;(\eta_{m-h}=95\%)\;$. The generated heat energy is assumed to be distributed by conduction to the grinding tool (*Q_t_*), the workpiece (*Q_w_*), and the chips (*Q_c_*), while neglecting the portion transferred to the environment by convection and radiation. The portion of total generated heat conducted to the machined through the contact surface $A_{w}$ of relative location $\left(x_{j}\right)$ can be determined as:

(5) $$Q_{w}\left(x_{j}\right)=\frac{A_{w}\cdot k_{w}}{TDD}\cdot \left(\;T_{x_{j}}-T_{0}\right)$$

In Equation (5), we note that the *TDD* values (already determined in Table 5) are permuted between the couples of specimens (B/C) and (D/E) while keeping the *TDD* value of the pure matrix work part A. The reason for this consideration is that the thermal diffusion phenomenon, which is related to the fiber orientation, in the workpiece depth direction ($\vec{y}$) occurs inversely to that of the machining direction ($\vec{x}$). The thermal conductivity value $k_{w}\;$ of each workpiece is estimated by weighting the ingredient volume proportions in Table 3. The heat energy conducted to the tool can be calculated by the thermal capacity formula:

(6) $$Q_{t}=m_{t}\cdot C_{pt}\cdot \left(\;T_{max}-T_{0}\right)$$

Here, the mass of the tool and its specific capacity are: *m_t_ =* 16 × 10^−3^ kg, and *C_pt_ =* 492 J∙kg^−1^∙K^−1^. Afterwards, the heat partition ratio (*R_t_*, *R_w_*, and *R_c_*) of each conducted portion of total heat can be deduced according to Equations (7)–(9):

(7) $$R_{t}=\frac{Q_{t}}{Q_{Total}}$$

(8) $$R_{w}=\frac{Q_{w}}{Q_{Total}}$$

(9) $$R_{c}=\frac{Q_{c}}{Q_{Total}}=\frac{Q_{Total}-Q_{t}-Q_{w}}{Q_{Total}}$$

The values of the distributed heat energies and their heat partition ratios are numerically calculated from Equations (4)–(9) and regrouped in Table 7. The last column of this table contains the standard deviation (SD) of the obtained results.

Table 6 shows that over 60% of the heat generated in the edge grinding process is conducted into the work parts. However, a smaller heat partition is dissipated through the metal diamond tool. This is generally due to the difference of heat capacity between the specimens, on the one hand, and the grinding tool, on the other. Although the metal diamond tool is a good heat conductor, its low quantity of matter does not store as much energy as that of the tested specimens [29]. Furthermore, it appears that silica sand grains play a very important role in increasing heat conductivity of the GFRP material against reducing that transmitted to the grinding tool and authorizing more heat (with an amount of 20% for 0° fiber orientation and 10% for perpendicular configuration) to be absorbed within the microstructure workpiece [45]. In the same context, Figure 7 shows that the silica sand filler contributes to keeping the tool temperatures down to an admissible level. Concerning the fiber orientation, it is shown that parallel fibers allowed less heat to be dissipated through the tested specimens. Contrariwise, the perpendicular orientation of reinforcement glass fibers contributes to more thermal energy generation by both the tool and the GFRP samples. The heat partition through the chips does not seem to remove more than 4% of the overall heat flux, and most of the generated heat in the edge grinding of GFRP with low depth of cut is dissipated through the work part and the diamond tool.

In order to end up at significant and objective interpretations, the results of the above heat partition discussion should be further confirmed and proven in the light of visual inspections carried out on scanning electron microscopy (SEM) images.

### 3.4. Scanning Electron Microscopy Investigation

To assess the effects of reinforcement fiber orientation and presence of silica sand filler on the quality of the GFRP edge milled surface and its state of damage, the machined surface SEM images of the five types were obtained. The samples were carefully prepared, and micrographs were captured upon Scanning Electron Microscope Model TS Qanta 250. SEM images were captured with a highest magnification level of 600, and saved with a resolution of 2048 × 1536 pixels at an accelerating voltage range of [10,25] kV, and under the low vacuum mode with a constant pressure of 70 pa [37].

#### 3.4.1. Edge Trimming-Induced Signature into Pure Epoxy Matrices

**Figure 8:**
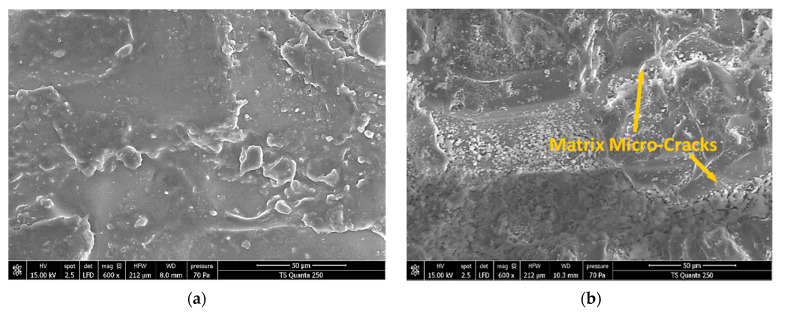
SEM Micrographs obtained before and after edge trimming pure epoxy matrices (sample A). (**a**) Pure epoxy (before machining). (**b**) Machined pure epoxy-f = 50 mm∙min^−1^.

When comparing SEM micrograph shots of the pure epoxy sample before and after edge trimming (Figure 8), it can be seen that the epoxy fresh cut area manifests much more irregular topography than that captured at its initial state [46]. The inspections reveal that the surface integrity of the machined pure epoxy edge is affected by the presence of several micro-cracks spread around the tridimensional profiles. This indicates that the development of such a failure signature depends strongly on the matrix thermal properties.

The mechanical properties of the material are also assumed to be slightly changed taking into account that the peak temperature ranges from 0.18 *T_g_* to 0.26 *T_g_*. Crack development might as well refer to residual stresses raised by trimming [47].

#### 3.4.2. Effects of Silica Sand Filler in Edge Trimming-Induced Signature into 0° Fiber Orientation Materials

**Figure 9:**
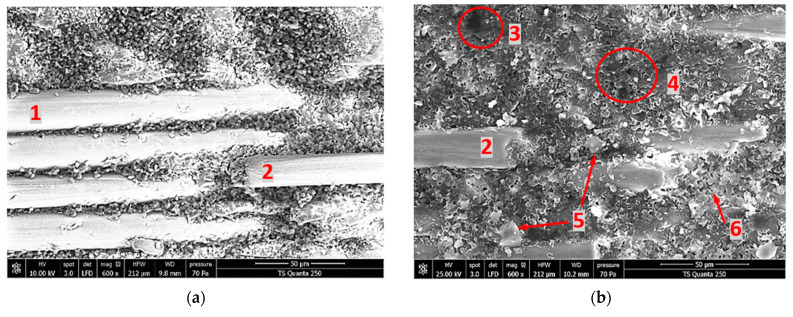
SEM Micrographs obtained when edge-trimming TCP composites parallel to fiber orientation. **Legend**: (**1**) uncovered fiber, (**2**) fractured fiber, (**3**) unfilled hiatus, (**4**) filled hiatus, (**5**) milled silica grains, (**6**) matrix debris. (**a**) Specimen B. (**b**) Specimen D.

For the purpose of determining the effects of the silica sand filler on surface integrity and process-induced damage, fresh surface analyses were performed on 0° reinforcement fiber orientation Glass/Epoxy composites with and without silica sand grains. The obtained cut area micrographs after edge-trimming of specimens B and D are illustrated in Figure 9a,b, respectively. Random measurement inspections on magnified view allow the rough determination of the diameters of uncovered fiber on specimens’ trimmed area. From the micrograph shots, the arithmetic average of diameters was found to be about 20 μm.

From inspections, it is clear that the fibers in sample B maintain regular diameters, unlike those of sample D, which appear to be split. Indeed, the cut land of non-loaded work part manifests the presence of fiber and epoxy debris that constitute the cut chips embedded in the matrix and inside the interstices between fibers. In the case of the loaded specimen, residual chips appear more heterogeneous and of flatter shapes. Moreover, cut land of loaded samples contains several distributed hiatuses, which represents the old locations of silica sand grains already extracted during edge trimming. Once the silica grain is being extracted from the specimen, the metal bond tool acts to trundle it along the released material by inertial and gripping effects. The separated particles act in turn to fracture the glass fiber and to grind the material matrix. Thus, the material removal rate in this case is not only subjected to the cutting characteristics of abrasive grains upon the grinding wheel tool and the CNC machine setting but also to the mass and volume of silica sand grains. Otherwise, the glass fiber orientation in the cutting area is a strong process-induced damage sign. In specimen B, the fibers remain parallel while they appear to be splintered arbitrarily in the cut area of specimen D. For the two composite configurations, the fine chips of dusty nature are spread into the wheel tool trough and the cavities and grooves left by the filler particle displacement. The accumulation of this dust as machining progresses prevents the direct thermal conduction between the tool and composite material. Therefore, the thermal generation process seems to switch from tool-material to chips-matrix interfaces. Hence, the thermal and tribology characteristics of the dusted chips (which regenerates through the tool–part contact surface) play a significant role in the thermal behavior of TCP machining. At this stage, the tool temperature measurements obtained from infrared analysis can give strong indications about the level of tool thermal energy dissipation during machining based on the nature of the chips detected by the SEM analyses [48,49,50]. Consequently, it can be deduced that SEM and infrared analysis are complementary. Contrary to infrared captures, microscopic images cannot be obtained in real time of machining, but they allow us to distinguish defects undetectable by thermographic analysis.

#### 3.4.3. Effects of Silica Sand Filler in Edge Trimming-Induced Signature into 90° Fiber Orientation Materials

**Figure 10:**
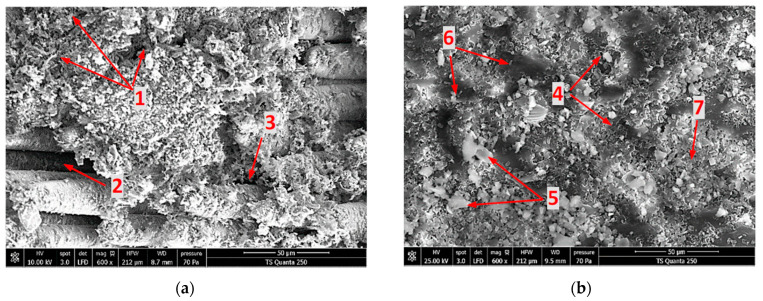
SEM Micrographs obtained when edge trimming of TCP composites perpendicular to fiber orientation. **Legend**: (**1**) Matrix cracking, (**2**) Interface failure, (**3**) Matrix loss, (**4**) Cut-section of GF, (**5**) Milled silica grains, (**6**) Digged area, (**7**) Matrix debris. (**a**) Specimen C. (**b**) Specimen E.

With the aim of examining the thermal damage induced during milling of TCP composites perpendicular to fiber orientation, SEM analyses were executed on specimens C and E. Figure 10 shows the obtained micrographs of fresh surfaces.

The results from the highly magnified SEM images help to draw the following observations:

- For **specimen C**, Figure 10a shows a catastrophic failure, since serious loss of epoxy matrix occurs around the fiber units, causing deep damage to the cutting area. In epoxy-covered zones, matrix-cracking propagations can be distinguished. Theses fissures likely result from thermal effects as peak temperatures reach their highest values at this configuration. Yet, mechanical effects can contribute to the propagation of these cracks through the influence of the pressure difference, which is caused by the tool’s forward movement between the upstream and downstream contact surfaces [47]. As the tool moves forward, the glass fibers bend under the action of the tool thrust force and then split by shearing. According to this cutting mechanism, the high rise of generated heat was due to the increase in frictional force between the composite reinforcement fibers and the tool, which in turn was due to the increase of the thrust force. The tool temperature curve (Figure 7) built from the infrared measurements is consistent with this assumption since the released thermal energy which can be estimated from the curve by numeric integration should be equal to the work of the frictional force. This is confirmed by the results obtained by Henerichs et al. [40], who examined, through SEM images, tool wear after machining of 90° CFRP fiber direction and observed an intensive tool wear compared to 0° fiber direction part. From Figure 10a, no remarkable fiber fragment was detected on the cut area, and the observed free residues go back potentially to pure matrix dusty chips. Unlike the cutting parallel to fiber process, perpendicular orientation leads to a neat fracture of glass fibers, resulting in dusty chips with a relatively uniform long size.
- For **specimen E**, the micrograph exhibited no significant matrix cracking. Despite the presence of random fluted zones caused by silica sand grain displacement, the cut area seems to be more consolidated. This improvement in mechanical properties is attributed to the increase of the *Tg* of filled samples since many publications [43,44,45] have affirmed that adding filler to the epoxy matrix can improve the thermal properties of composites, especially the glass transition temperature. Indeed, temperature drop obtained when adding silica sand fillers into 90° composite material laminates indicates the significant role of the filler grain in the heat generation mechanism. Silica grains act, by way of high thermal capacity, as thermal walls that limit heat transfer. From inspections, excavated areas likely resulted from silica grain movement. In addition, heterogeneous debris can be distinguished, including a milled epoxy resin–silica sand admixture. In particular, free silica milled grains potentially released from interfaces with an average grain size of eight microns were detected at the trim plan. Globally, the subsurface damage of specimen E appears less critical than that developed in Specimen C.

## 4. Conclusions

An experimental study of temperature histories was performed during dry edge trimming of GFRP composites. The analysis of the obtained results from SEM and thermographic inspections demonstrated reasonable qualitative and quantitative links between the composite lamination configuration and the machining generated heat. It was found that machining of silica sand-free GFRP parallel to fiber did not affect the temperature evolution if compared to that of pure epoxy polymer. SEM-enlarged images highlighted a major loss of epoxy resin covering the fiber filament units that can favor a fast degradation of the composite part. However, machining GFRP perpendicular to fiber acts to involve a sharp increase of peak temperature due to the important rise of friction, owing to rough and irregular cut fiber surfaces acting to localize pressure at the contact area. SEM capture revealed a catastrophic surface finish, including matrix loss, cracking, and interface failures. Thermographic inspections manifested a very high rise of tool temperature magnitude. In contrast, the addition of silica sand grains as filler reveals high reliability in terms of substantially changing the heat balance within the tool/material pair by reducing both tool and workpiece temperatures, especially when machining perpendicular to fiber. SEM-magnified images exhibited more consolidated fresh cut areas with fewer machining defects—i.e., matrix cracking and interface failures. Provided that cutting conditions were omitted in the design of the experiment to focus strictly on the effects of material properties on heat generation, an investigation of process parameters with the aim of inspecting the optimum machining conditions to be considered for the composite manufacturing layout becomes more difficult. In particular, the appropriate percentage of silica fillers must be rigorously addressed.

## Figures and Tables

**Table 1:** Specimens manufacturing configurations and characteristics.

Specimens	Ingredients Volume Fractions	Physical Characteristics
**A**	Epoxy: **100%**	k_A_ = 0.250 W∙m^−1^∙K^−1^ ρ_A_ = 1200 kg∙m^−3^
**B**	Epoxy: **85%** Glass fiber: **15%** Fiber orientation: **0°**	k_B_ = 0.217 W∙m^−1^∙K^−1^ ρ_B_ = 1410 kg∙m^−3^
**C**	Epoxy: **85%** Glass fiber: **15%** Fiber orientation: **90°**	k_C_ = 0.217 W∙m^−1^∙K^−1^ ρ_C_ = 1410 kg∙m^−3^
**D**	Epoxy: **57%** Glass fiber: **15%** Silica Sand filler: **28%** Fiber orientation: **0°**	k_D_ = 0.640 W∙m^−1^∙K^−1^ ρ_D_ = 1816 kg∙m^−3^
**E**	Epoxy: **57%** Glass fiber: **15%** Silica Sand filler: **28%** Fiber orientation: **90°**	k_E_ = 0.640 W∙m^−1^∙K^−1^ ρ_E_ = 1816 kg∙m^−3^

**Table 2:** Typical properties of liquid considered resins.

Property/Resin	Epoxy 1050
Appearance	Opalescent neutral liquid
Density	1.17
Viscosity (centipoise)	1043
Gel time (minutes)	210 (at 23 °C with the hardener 1055 s)

**Table 3:** Characteristics of silica grains.

Properties	Values
Appearance	Opalescent neutral liquid
Density	265 g/cm³
Molecular weight	60.1
Granulometry	0.3~0.8 mm
SiO_2_%	approx 99%
Grain shape	sub-angular

**Table 4:** Technical characteristics of data acquisition instruments [37].

Components	Specifications
8 Max6675 TCs Type K Sensor Module For Arduino	Voltage: 5V DC Intensity: 50 mA Accuracy: ±1.5 °C Resolution: 0.25 °C
Arduino board based on ATMega2560 with USB connection	16 MHz 54 Inputs/Outputs including: 14 PWM (Pulse Width Modulation) input channels, 16 analog input channels, and, 4 UART (Universal Asynchronous Receiver-Transmitter) channels
Dupont male/female connection cables	Suitable cable model
Computer with Arduino user interface	Suitable interface

**Table 5:** Summary of sampling points heating rates (°C∙s^−1^) and thermal diffusion distances (mm) along the manifesting region.

		TC1	TC2	TC3	TC4	TC5	TC6	TC7	TC8
A	${\dot{T}}_{h}$	0.191	0.273	0.309	0.416	0.433	0.618	0.585	0.733
	*TDD*	7	6.5	5.5	6	6	5.5	6.5	6
	${\dot{T}}_{c}$	0.0142	0.0152	0.0179	0.0279	0.0320	0.0384	0.0439	0.0483
B	${\dot{T}}_{h}$	0.300	0.315	0.466	0.615	0.600	0.463	0.427	0.544
	*TDD*	10	11	7.5	6.5	7	9.5	11	9
	${\dot{T}}_{c}$	0.030	0.034	0.045	0.047	0.053	0.058	0.068	0.071
C	${\dot{T}}_{heat}$	0.145	5.784	7.733	7.428	11.568	9.348	13.308	18.160
	*TDD*	5	3.7	3	3.5	2.5	3.5	2.6	2
	${\dot{T}}_{c}$	0.196	0.224	0.249	0.277	0.318	0.354	0.384	0.389
D	${\dot{T}}_{heat}$	0.155	0.186	0.158	0.240	0.512	0.354	0.401	0.472
	*TDD*	7.1	7	10	7.5	6.5	6.5	6	5.5
	${\dot{T}}_{c}$	0.017	0.019	0.022	0.026	0.029	0.035	0.043	0.039
E	${\dot{T}}_{heat}$	0.280	0.288	0.373	0.564	0.486	0.818	0.858	1.020
	*TDD*	7.5	9	7.5	5.5	7	4.5	4.5	4.5
	${\dot{T}}_{c}$	0.031	0.040	0.044	0.050	0.059	0.062	0.069	0.081

**Table 6:** Summary of heating rate measurements and correspondent R-square values.

*Specimen*	A	B	C	D	E
*V_H_* (°C∙s^−1^)	0.108	0.063	0.610	0.051	0.075
*R-square*	0.9627	0.9799	0.9837	0.9775	0.9837

**Table 7:** Energy partition and heat distribution ratios of edge grinding of the different GFRP lamination configurations.

Specimen	$Q_{Total}\;(J)$	$Q_{t}\;(J)$	$R_{t}$	$Q_{w}(J)$	$R_{w}$	$Q_{c}\;(J)$	$R_{c}$	SD (%)
A	314.429	86.592	0.275	215.260	0.684	12.577	0.039	0.57
B	834.046	245.606	0.294	539.575	0.646	24.865	0.0298	0.82
C	1961.424	566.784	0.286	1343.400	0.677	71.240	0.036	1.01
D	456.5	55.891	0.121	387.927	0.840	17.682	0.0383	1.14
E	751.082	138.547	0.182	593.510	0.782	26.025	0.0343	1.26

## Data Availability

Not applicable.
